# Clinical and sonographic characteristics of gastric-type endocervical adenocarcinoma: a descriptive case series of 13 patients

**DOI:** 10.3389/fmed.2026.1932627

**Published:** 2026-10-28

**Authors:** Tiezheng Li, Meiping Jiang, Yingchun Luo, Yu Long

**Affiliations:** Ultrasound Medicine Center, The Affiliated Hospital of Maternal and Child Health Care, Hengyang Medical School, University of South China, Changsha, Hunan Province, China

**Keywords:** gastric-type endocervical adenocarcinoma, honeycomb pattern, HPV-independent cervical adenocarcinoma, sonographic features, ultrasonography

## Abstract

**Objective:**

Gastric-type endocervical adenocarcinoma (G-EAC) is a distinct human papillomavirus (HPV)-independent subtype of cervical adenocarcinoma for which early diagnosis continues to pose a clinical challenge. We set out to characterize the clinical and sonographic features of G-EAC and to provide hypothesis-generating observations to guide future research.

**Methods:**

This retrospective analysis comprised 13 patients with histopathologically confirmed G-EAC who underwent surgery at our center between August 2017 and September 2024. We reviewed and descriptively summarized the clinical presentation, HPV and cytology results, cervical biopsy findings, tumor markers, and ultrasonographic and cross-sectional imaging findings, together with all available pathological and surgical records.

**Results:**

The patients were aged 39–76 years (mean, 53.4 years), and all were parous. Vaginal discharge was the most common presenting symptom (8/13, 61.5%), followed by irregular vaginal bleeding (4/13, 30.8%) and postcoital bleeding (1/13, 7.7%). HPV testing was negative in 11/13 patients (84.6%); 1/13 (7.7%) tested positive for HPV 58, and another 1/13 (7.7%) carried a different high-risk HPV type. ThinPrep cytologic testing (TCT) was negative in 12/13 patients (92.3%), whereas atypical squamous cells were detected in 1/13 (7.7%). Among the 11 patients who underwent colposcopy with cervical biopsy, histopathological findings were negative in 8/11 (72.7%), mild glandular atypia was identified in 2/11 (18.2%), and rare adenocarcinoma cells were identified in 1/11 (9.1%). All 13 patients underwent preoperative ultrasound. Ultrasound findings raised suspicion of cervical malignancy in 9/13 patients (69.2%), were suggestive of benign cervical cystic lesions in 2/13 (15.4%), and were unremarkable in the remaining 2/13 (15.4%). A honeycomb sonographic pattern was present in 7/13 patients (53.8%), and abundant intralesional vascularity (Adler grade III) was present in 9/13 patients (69.2%). Preoperative MRI was performed in 10/13 patients (76.9%), 1 of whom also underwent CT, whereas the remaining 3/13 patients (23.1%) underwent neither MRI nor CT. A barrel-shaped cervix was documented in the pathological or surgical records of 4/13 patients (30.8%).

**Conclusion:**

G-EAC may come to attention through a combination of clinical and imaging findings that together raise diagnostic suspicion. In this series, HPV negativity was common, and vaginal discharge was the most frequent presenting complaint. A honeycomb sonographic pattern and abundant intralesional vascularity may raise suspicion of G-EAC, although these findings remain descriptive and require validation in larger, adequately controlled studies.

## Introduction

Gastric-type endocervical adenocarcinoma (G-EAC) is an HPV-independent subtype of cervical adenocarcinoma and the second most common type after usual-type endocervical adenocarcinoma (1). Histologically, G-EAC shows gastric differentiation, with morphologic features resembling those of pyloric gland epithelium. The tumor often arises in the endocervical canal and may lie deep within the cervical stroma, thereby limiting the yield of routine cytologic screening and superficial biopsy. Although the histologic appearance of G-EAC may mimic that of benign glandular lesions, the tumor behaves aggressively, and these features may contribute to diagnostic delay and unfavorable clinical outcomes (2).

Although a barrel-shaped cervix and a honeycomb sonographic pattern have been described in earlier imaging series, data on the sonographic features of G-EAC remain limited and largely descriptive, particularly in cases with missed or incorrect preoperative ultrasound interpretations. We therefore set out to describe the clinical and sonographic features of 13 patients with pathologically confirmed G-EAC and to identify potential imaging findings that may warrant further investigation.

## Materials and methods

### General data

We retrospectively reviewed the clinical and imaging data of 13 patients with pathologically confirmed G-EAC who underwent surgery at our institution between August 2017 and September 2024. All patients were parous women aged 39–76 years (mean age, 53.4 years), and all 13 underwent preoperative transvaginal ultrasound. Preoperative MRI was performed in 10 patients, 1 of whom also underwent CT; the remaining 3 underwent neither modality. The diagnosis of G-EAC was established in accordance with the 2020 World Health Organization (WHO) Classification of Tumours of Female Reproductive Organs. The inclusion criteria were as follows: (1) surgical treatment at our institution during the study period; (2) histopathological confirmation of G-EAC from biopsy, conization, or surgical specimens according to the 2020 WHO Classification; and (3) complete preoperative ultrasound data. Exclusion criteria were as follows: (1) receipt of neoadjuvant chemotherapy or radiotherapy before surgery; and (2) incomplete clinical or imaging data. Eligible patients were retrieved from the institutional pathology database. The follow-up cutoff was August 2026. The study protocol was approved by the Hospital Ethics Committee (Approval No. 2025-SY-096). Because of the retrospective design, the requirement for written informed consent was waived. All patient data and images were fully anonymized before analysis.

## Equipment and methods

### Equipment

Examinations were performed with GE Voluson E10, Samsung HERA W10, and Mindray Resona 8 color Doppler ultrasound systems and endocavity probes operating at 5–10 MHz.

### Examination methods

All 13 patients underwent preoperative transvaginal ultrasound. Patients were placed in the lithotomy position, and the uterus and bilateral adnexa were scanned systematically in multiple planes. When a cervical lesion was identified, its size, location, margins, and internal echogenicity were recorded. Intralesional and perilesional vascularity was assessed with color Doppler imaging. We categorized vascularity descriptively using the Adler grading framework (3): grade 0, no detectable color flow signals; grade I, one or two punctate persistent flow signals within the tumor (diameter <0.1 cm); grade II, multiple small vessels or a single dominant vessel; grade III, four or more distinct vessels within the lesion. Because of the retrospective design, detailed Doppler acquisition parameters (pulse-repetition frequency, color gain, and wall-filter settings) were not uniformly archived and could not be standardized. Tumors were staged according to the 2018 International Federation of Gynecology and Obstetrics (FIGO) staging system for cervical cancer.

### Ultrasound definitions

To standardize the reporting of sonographic findings, we adopted the following operational definitions: (1) a honeycomb pattern was defined as a heterogeneous solid-appearing area containing multiple small anechoic spaces, consistent with the microcystic architecture reported in gastric-type mucinous lesions (4, 5); and (2) abundant vascularity was defined as Adler grade III, namely four or more distinct intralesional color flow signals (3). These definitions were applied for descriptive purposes only and should not be interpreted as validated diagnostic criteria for G-EAC. A barrel-shaped cervix was recorded separately whenever it was explicitly documented in the pathology or surgical records.

#### Image interpretation

All ultrasound images were reviewed retrospectively by two sonographers, each of whom had more than 5 years of experience in gynecological sonography. Any disagreements were resolved through consensus. The two reviewers were not blinded to the final histopathological diagnosis. Preoperative ultrasound impressions were taken from the original ultrasound reports, whereas the sonographic features, including the honeycomb pattern and the vascularity, were assessed during the retrospective review of the images.

#### Statistical analysis

Only descriptive statistics are reported. Continuous variables are summarized as mean (range) and categorical variables as frequencies (percentages). No inferential testing was undertaken, given the small sample size and the absence of any control group.

#### Follow-up

Postoperative follow-up data were collected from institutional medical records, and each patient's status was recorded as ongoing, lost to follow-up, or deceased.

## Results

### Clinical data

Vaginal discharge was the most common presenting symptom among the 13 patients with G-EAC (8/13, 61.5%), followed by irregular vaginal bleeding (4/13, 30.8%) and postcoital bleeding (1/13, 7.7%). HPV testing was negative in 11/13 patients (84.6%); 1/13 (7.7%) tested positive for HPV 58, and another 1/13 (7.7%) tested positive for a different high-risk HPV type. TCT was negative in 12/13 patients (92.3%), and atypical squamous cells were detected in the remaining 1/13 (7.7%). Tumor markers were elevated in only a minority of patients: CA19-9 in 4/13 (30.8%), CA125 in 4/13 (30.8%), CEA in 2/13 (15.4%), and SCC antigen in 2/13 (15.4%). Among the 11 patients who underwent colposcopy with cervical biopsy, histopathological findings were negative in 8/11 (72.7%), mild glandular atypia was present in 2/11 (18.2%), and rare adenocarcinoma cells were present in 1/11 (9.1%). Table 1 summarizes the clinical characteristics of these 13 patients.

**Table 1:** Clinical characteristics of 13 patients with G-EAC.

No.	Age (years)	Clinical presentation	Tumor markers				HPV	TCT	Imaging findings	
			CA19-9 (U/mL)	CA125 (U/mL)	CEA (ng/mL)	SCC (μg/L)			Ultrasound	MRI/CT
1	54	Irregular vaginal bleeding for >2 months	102	42.2	<5	<1.5	–	–	Endometrial cavity fluid	MRI: Cervical thickening, multiple cystic lesions in cervix
2	54	Vaginal discharge for >20 years, increased leucorrhea for 1 month	<35	<35	<5	<1.5	–	–	Cervical lesion: highly suspicious for malignancy; Endometrial cavity fluid	MRI + CT: Irregular cervical mucosa, Nabothian cysts
3	65	Postmenopausal vaginal bleeding	202.2	<35	180.1	<1.5	–	–	No cervical abnormality detected	MRI: Cervical mass: suspected cervical cancer
4	76	Postmenopausal vaginal discharge for >10 years	<35	<35	10.2	<1.5	–	–	Cervical lesion: highly suspicious for malignancy	MRI: Cervical mass: mucinous adenoma (minimal deviation adenocarcinoma)?
5	46	Postcoital bleeding for >1 year	<35	<35	<5	1.7	HPV58 positive	Atypical squamous cells	Irregular cervical enlargement with abnormal echogenicity, suspicious for malignancy; Endometrial cavity fluid	MRI: Anterior cervical wall mass: suspected cervical cancer
6	45	Vaginal discharge for >8 years	<35	<35	<5	<1.5	–	–	Cervical lesion: highly suspicious for malignancy	MRI: Multilocular cystic mass in cervix
7	55	Intermittent vaginal discharge for 3 years	<35	122.2	<5	<1.5	–	–	Endocervical nabothian cyst	/
8	48	Intermittent vaginal discharge for 10 years	<35	<35	<5	<1.5	–	–	Endocervical cyst with possible infection	MRI: Slightly enlarged cervix, irregular nodular shadow in cervix
9	47	Vaginal discharge for >7 years	113	40.2	<5	<1.5	Other high-risk (one of 11 high-risk types)	–	Cervical hypertrophy with abnormal echogenicity: highly suspicious for malignancy	/
10	45	Irregular vaginal bleeding	<35	<35	<5	1.9	–	–	Cervical abnormality: suspicious for cervical cancer	MRI: Cervical mass: suspected cervical cancer
11	60	Postmenopausal vaginal discharge for >4 months	<35	<35	<5	<1.5	–	–	Cervical abnormality: suspicious for cervical cancer	MRI: Cervical cancer (Stage IIB)
12	60	Postmenopausal vaginal discharge	479.6	51.9	<5	<1.5	–	–	Cervical abnormality: suspicious for cervical cancer; Endometrial cavity fluid	/
13	39	Mild irregular vaginal bleeding	<35	<35	<5	<1.5	–	–	Cervical abnormality: suspicious for cervical cancer; Endometrial cavity fluid	MRI: Cervical cancer (Stage IB)

CA19-9, carbohydrate antigen 19-9; CA125, carbohydrate antigen 125; CEA, carcinoembryonic antigen; SCC, squamous cell carcinoma antigen. “–” indicates a negative HPV/TCT result, whereas “/” indicates that MRI/CT was not performed.

### Ultrasound findings

Retrospective review of the ultrasound images revealed heterogeneous sonographic appearances across the 13 patients with G-EAC. A honeycomb pattern was observed in 7/13 patients (53.8%) and consisted of heterogeneous solid-appearing areas containing multiple small anechoic spaces. Among the remaining patients, 2/13 (15.4%) had cervical anechoic areas with poor through-transmission and ill-defined margins; 1/13 (7.7%) showed an anechoic area containing intermediate-level echoes; 1/13 (7.7%) had multiple well-defined cervical anechoic foci with posterior acoustic enhancement; and 2/13 (15.4%) showed no significant abnormality in cervical echogenicity. Endometrial cavity fluid was also observed in 5/13 patients (38.5%). Color Doppler assessment identified Adler grade 0 vascularity in 2/13 patients (15.4%), grade II in 2/13 (15.4%), and grade III in 9/13 (69.2%); no patient had grade I vascularity. The preoperative ultrasound reports raised suspicion of cervical malignancy in 9/13 patients (69.2%), were suggestive of benign cervical cystic lesions in 2/13 (15.4%), and reported no cervical abnormality in 2/13 (15.4%). These proportions are descriptive findings from a single case series and should not be interpreted as estimates of sensitivity, specificity, or predictive value, since no control group was included. The sonographic findings of the 13 patients are summarized in Table 2, and representative images of four distinct patterns are shown in Figure 1 (Cases 4, 5, 6, and 11).

**Table 2:** Ultrasound findings in 13 patients with gastric-type endocervical adenocarcinoma (G-EAC).

No.	Cervical morphology	Cervical echogenicity	Lesion size (cm)	Lesion margin	CDFI	Adler grade	Endometrial cavity/endometrium	Ultrasound impression
1	Normal cervical size, normal endocervical structure	Homogeneous myometrial echogenicity	No abnormality	No abnormality	No obvious abnormal blood flow signals in cervix	Grade 0	1.5 cm anechoic area in mid-upper uterine cavity	Endometrial cavity fluid
2	Markedly enlarged cervix, abnormal endocervical structure	Thin cervical stroma, heterogeneous slightly hyperechoic areas with multiple anechoic zones	6.2 × 6.0 × 6.8	Ill-defined	Multiple punctate and linear blood flow signals in cervix	Grade III	1.1 cm anechoic area in mid-upper uterine cavity	Cervical lesion: highly suspicious for malignancy; Endometrial cavity fluid
3	Normal cervical size, normal endocervical structure	Homogeneous myometrial echogenicity	No abnormality	No abnormality	Linear color flow signals in cervix	Grade II	Homogeneous endometrium	No cervical abnormality
4	Normal cervical size, abnormal endocervical structure	Irregularly thickened mucosa, slightly hyperechoic, heterogeneous, with multiple small anechoic areas	3.3 × 1.8 × 3.0	Ill-defined	Multiple punctate and linear blood flow signals in cervix	Grade III	Homogeneous endometrium	Cervical lesion: highly suspicious for malignancy
5	Irregularly enlarged cervix, abnormal endocervical structure	Thickened mucosa, slightly coarse and hyperechoic myometrial echoes, hypoechoic area with anechoic spaces in mid-lower anterior wall	1.7 × 0.9 × 1.3	Ill-defined	Multiple punctate and linear blood flow signals within lesion	Grade III	0.48 cm anechoic area in mid-upper uterine cavity	Irregular cervical enlargement with abnormal echogenicity, suspicious for malignancy; endometrial cavity fluid
6	Irregularly enlarged cervix, abnormal endocervical structure	Aggregated anechoic areas of varying sizes, “petal-like” appearance, some communicating	4.0 × 3.1 × 4.2	Partially ill-defined	Multiple punctate and linear blood flow signals in cervix	Grade III	Homogeneous endometrium	Cervical lesion: highly suspicious for malignancy
7	Normal cervical size, normal endocervical structure	Anechoic area in cervical stroma with posterior acoustic enhancement	1.2 × 1.2 × 1.1 (largest)	Well-defined	No obvious blood flow signals	Grade 0	Homogeneous endometrium	Endocervical nabothian cyst
8	Normal cervical size, normal endocervical structure	Anechoic area with mixed intermediate echoes within cervical stroma	1.5 × 1.2 × 1.1 (largest)	Partially ill-defined	Linear color flow signals within intermediate echo area	Grade II	Homogeneous endometrium	Endocervical cyst with possible infection
9	Markedly enlarged cervix, abnormal endocervical structure	Increased and coarse parenchymal echoes, multiple anechoic areas with dense weak internal echoes	4.0 × 1.6 × 2.2	Partially ill-defined	Multiple punctate and linear blood flow signals in cervix	Grade III	Homogeneous endometrium	Cervical hypertrophy with abnormal echogenicity: highly suspicious for malignancy
10	Normal cervical size, obliterated endocervical structure	Hypoechoic area with multiple anechoic spaces	3.5 × 3.9 × 4.3	Ill-defined	Relatively abundant linear color flow signals within lesion	Grade III	Homogeneous endometrium	Cervical abnormality: suspicious for cervical cancer
11	Irregularly enlarged cervix, abnormal endocervical structure	Hyperechoic area with multiple anechoic spaces	2.7 × 2.3 × 2.1	Ill-defined	Relatively abundant linear color flow signals within lesion	Grade III	Homogeneous endometrium	Cervical abnormality: suspicious for cervical cancer
12	Enlarged cervix, abnormal endocervical structure	Hyperechoic area with multiple anechoic spaces	4.2 × 2.6 × 4.1	Ill-defined	Relatively abundant linear color flow signals within lesion	Grade III	0.7 cm anechoic area in mid-upper uterine cavity	Cervical abnormality: suspicious for cervical cancer; Endometrial cavity fluid
13	Normal cervical size, obliterated endocervical structure	Hypoechoic area with multiple anechoic spaces	1.3 × 1.0 × 1.2	Ill-defined	Relatively abundant linear color flow signals within lesion	Grade III	0.5 cm anechoic area in mid-upper uterine cavity	Cervical abnormality: suspicious for cervical cancer; Endometrial cavity fluid

CDFI, color doppler flow imaging.

**Figure 1:**
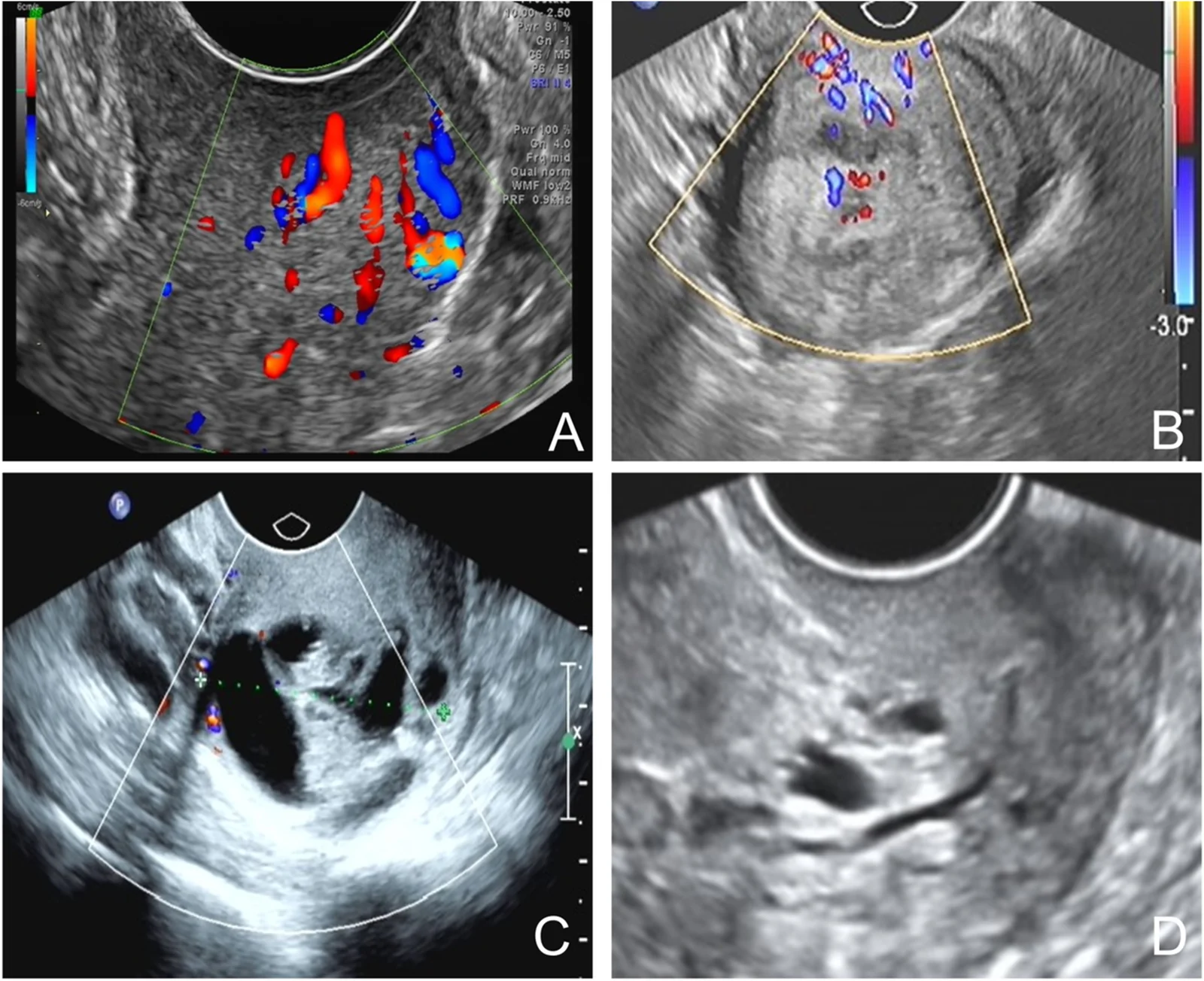
Representative ultrasound images of G-EAC from four patients (Cases 4, 5, 6, and 11). **(A)** Case 4 showed a normal-sized cervix with abnormal endocervical structure and heterogeneous echogenicity containing multiple small anechoic spaces, forming a “honeycomb” pattern; color Doppler imaging showed abundant intralesional blood flow (Adler grade III). **(B)** Case 5 showed irregular cervical enlargement with thickened, coarse, hyperechoic mucosa and a hypoechoic area containing anechoic spaces in the mid-lower anterior wall; color Doppler imaging showed abundant intralesional blood flow (Adler grade III). **(C)** Case 6 showed irregular cervical enlargement with aggregated anechoic areas of varying sizes, some of which communicated with one another to produce a “petal-like” appearance; color Doppler imaging showed abundant intralesional blood flow (Adler grade III). **(D)** Case 11 showed irregular cervical enlargement with hyperechoic areas containing multiple anechoic spaces in a “honeycomb” pattern.

### Pathological findings

Pathological findings among the 13 patients were as follows: stromal invasion depth was recorded in 11 patients and was <1/3 in 1/11 (9.1%), 1/3–2/3 in 3/11 (27.3%), and >2/3 or full-thickness in 7/11 (63.6%). Stromal invasion depth was unavailable in Cases 9 and 13. Vaginal involvement was present in Cases 5 and 10, whereas pathological assessment of vaginal involvement was unavailable or not assessable in Cases 1, 11, and 13. Lymphovascular space invasion (LVSI) was detected in 4/13 patients (30.8%). A barrel-shaped cervix was documented separately in the pathology or surgical records of 4/13 patients (30.8%), but this feature was not systematically assessed with ultrasound in the present study. Immunohistochemical findings varied according to the markers recorded in the available pathological records; a common denominator was therefore not assumed across the individual markers. Representative imaging and histopathological findings of G-EAC are illustrated in Figure 2 (Case 2).

**Figure 2:**
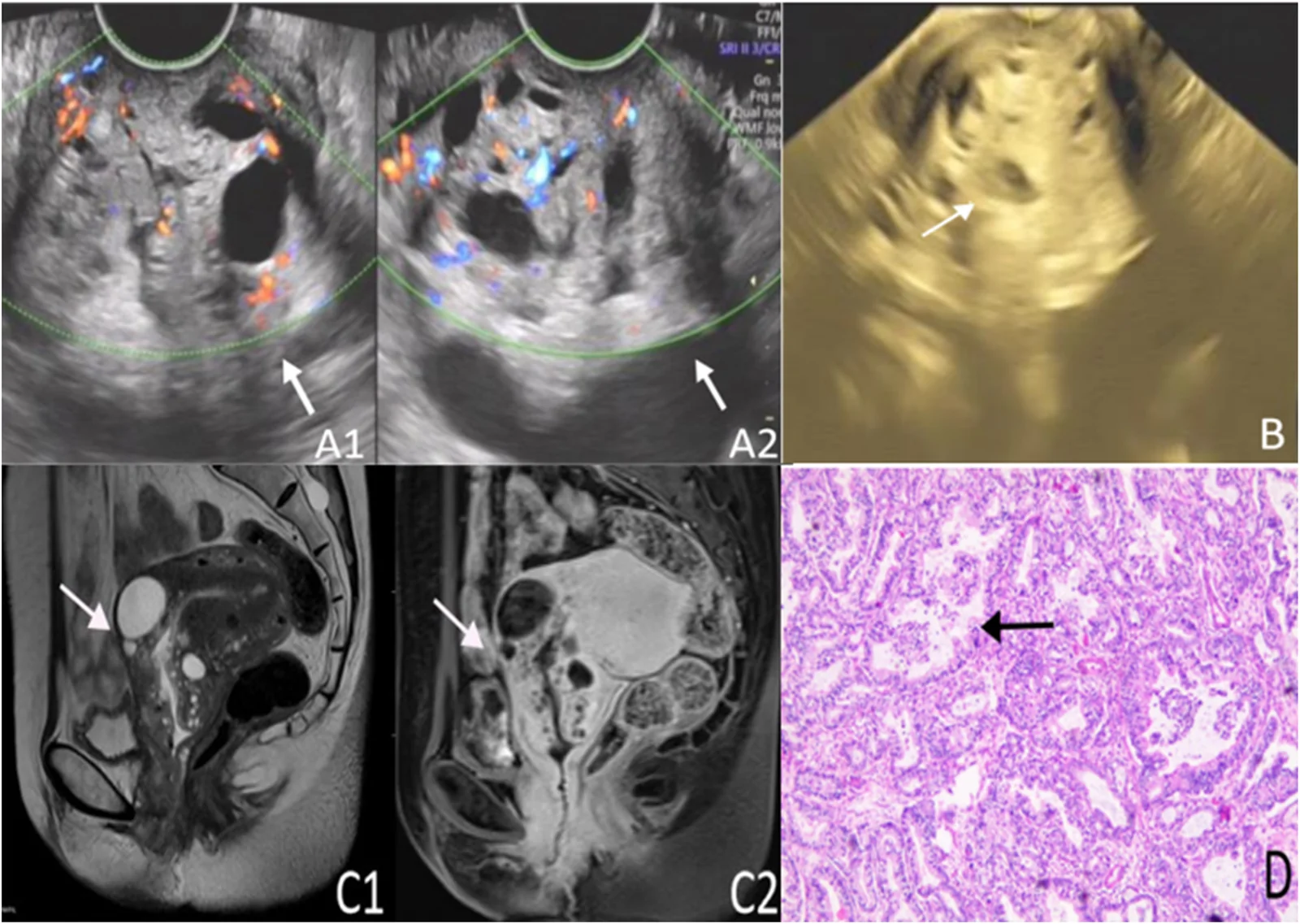
Representative imaging and histopathological findings in one patient with G-EAC (Case 2). **(A1)** Color Doppler image showing abundant intralesional blood flow (Adler grade III; white arrows indicate the color flow signals). **(A2)** Color Doppler image superimposed on the grayscale image, showing a diffusely enlarged cervix with heterogeneous echogenicity (white arrows indicate the heterogeneous cervical parenchyma). **(B)** Three-dimensional ultrasound image of the cervix showing diffuse cervical enlargement and cystic spaces. **(C1)** T2-weighted MRI sequence showing multiple round-like cystic lesions of varying sizes in the cervical canal, with hyperintense signals on T2-weighted images and smooth cystic walls; **(C2)** contrast-enhanced MRI showing multiple cystic lesions in the cervical canal, without enhancement of the fluid components. **(D)** Histopathological image showing glands of diverse morphology, with irregularly arranged glandular and cribriform structures and intraluminal papillary projections (H&E staining, ×40).

### Postoperative follow-up outcomes

Table 3 summarizes postoperative follow-up outcomes for the 13 patients. During follow-up, ten patients remained under routine surveillance and two were lost to follow-up; one died of unrelated cardiovascular and cerebrovascular diseases. Detailed data for each patient are provided in the same table.

**Table 3:** Clinicopathological characteristics, treatment, and postoperative follow-up outcomes of 13 patients with gastrictype endocervical adenocarcinoma (G-EAC).

No	FIGO stage	Surgery	Pathological tumor size (cm)	Adjuvant treatment	Follow-up status	Notes
1	IA2	CKC + ECC	–	None	Ongoing follow-up	—
2	IB3	RH + PLND	6.5 × 5.4 × 2.0	Ongoing chemoradiotherapy	Ongoing follow-up	—
3	IB2	RH + PLND	3.0 × 2.0 × 1.0	Completed scheduled chemoradiotherapy	Ongoing follow-up	—
4	IB1	RH + PLND	1.2 (diam)	—	Deceased	Death unrelated to G-EAC (cardiovascular/cerebrovascular disease)
5	IIA1	RH + PLND	3.5 × 3.0 × 2.5	None	Ongoing follow-up	—
6	IB3	RH + PLND	4.2 × 2.3 × 1.4	None	Ongoing follow-up	—
7	IB2	RH + PLND	1.5 × 3.0 × 2.1	None	Ongoing follow-up	—
8	IB1	RH + PLND	1.2 (diam)	None	Ongoing follow-up	—
9	IB3	RH + PLND	4.0 (diam)	None	Ongoing follow-up	—
10	IIB	RH + PLND	2.0 × 3.1 × 2.1	Completed scheduled chemoradiotherapy	Ongoing follow-up	—
11	IIB	RH + PLND	3.0 × 2.4 × 1.2	—	Lost to follow-up	—
12	IIIC2p	RH + PLND	4.0 × 2.4 × 2.0	—	Lost to follow-up	—
13	IB3	RH + PLND	4.1 × 3.7 × 5.1	Ongoing chemoradiotherapy	Ongoing follow-up	—

CKC, cold knife conization; ECC, endocervical curettage; RH, radical hysterectomy; PLND, pelvic lymphadenectomy; FIGO, international federation of gynecology and obstetrics; diam, diameter.

Tumor size is reported either as a maximum diameter (single value) or as three-dimensional measurements (length × width × height) taken from the pathology reports. Missing values are indicated as “—”.

## Discussion

Gastric-type endocervical adenocarcinoma (G-EAC) is an HPV-independent subtype of cervical adenocarcinoma with distinct clinicopathological characteristics. Because the tumor is HPV-independent, HPV-based screening may remain negative, and a deep location within the endocervical canal and cervical stroma can further limit its detection by cytology and superficial cervical biopsy (6–9). In this retrospective descriptive series of 13 patients with pathologically confirmed G-EAC, the combined clinical, sonographic, cross-sectional imaging, and pathological findings illustrate why this uncommon tumor may be difficult to recognize before surgery. Persistent symptoms despite negative conventional screening, together with suspicious cervical imaging abnormalities, should prompt consideration of a deeply located glandular lesion that warrants further evaluation.

### Clinical and screening features

In our series, most patients tested negative on HPV testing, cytology, and colposcopic biopsy. These findings are in keeping with the HPV-independent biology of G-EAC and illustrate the limitations of conventional cervical screening and superficial biopsy for detecting lesions that lie deep within the endocervical canal (6–9). Negative HPV testing, cytology, or superficial cervical biopsy do not necessarily exclude an occult glandular malignancy when clinical or imaging findings remain suspicious.

In our cohort, persistent vaginal discharge was the most frequent presenting symptom, followed by irregular vaginal bleeding and postcoital bleeding. Similar presentations have been reported in earlier studies of HPV-independent and gastric-type cervical adenocarcinoma (8, 10). Gastric-type and usual-type endocervical adenocarcinomas also differ in their clinicopathological and molecular characteristics (11). Because these symptoms are relatively nonspecific, they may not immediately suggest cervical malignancy, especially when routine cervical screening remains negative. Persistent or unexplained vaginal discharge or bleeding should likewise prompt further assessment when clinical examination or imaging findings raise concern about an underlying cervical lesion. In this series, tumor markers were elevated in only a minority of patients; given their nonspecific nature, they should be interpreted as adjunctive clinical information rather than as diagnostic findings.

### Sonographic characteristics and diagnostic pitfalls

Sonographic appearances of G-EAC varied considerably across this series. A honeycomb-like pattern, operationally defined as a heterogeneous solid-appearing area containing multiple small anechoic spaces, was present in 7/13 patients (53.8%), and abundant intralesional vascularity (Adler grade III) was seen in 9/13 patients (69.2%). This microcystic or multicystic appearance may reflect the underlying glandular architecture together with the mucin-containing spaces of gastric-type cervical lesions (4, 6). These findings should, however, be regarded as descriptive imaging clues rather than as specific diagnostic signs.

Not all G-EAC lesions showed an overtly malignant sonographic appearance. Although the original preoperative ultrasound reports raised suspicion of cervical malignancy in most of these patients, some were interpreted as benign cervical cystic lesions, whereas others showed no cervical abnormality. This variability highlights an important diagnostic pitfall in ultrasound. Lesions that are predominantly cystic or multicystic may resemble benign cervical conditions, whereas deeply located lesions, and those without marked cervical enlargement, may be difficult to identify on routine sonographic examination. The absence of an obvious solid mass or of other features conventionally regarded as malignant should not preclude consideration of G-EAC when clinical or imaging findings remain suspicious.

The mere presence of multiple cervical cystic spaces is by itself insufficient to distinguish G-EAC from benign multicystic cervical lesions. Nabothian cysts and lobular endocervical glandular hyperplasia (LEGH) may show overlapping imaging features, and gastric-type neoplastic lesions may themselves show variable proportions of solid and cystic components (12–14). Sonographic morphology should therefore be assessed in the context of lesion margins, solid components, vascularity, clinical presentation, and complementary imaging rather than as a stand-alone diagnostic feature.

Color Doppler showed abundant intralesional vascularity in a considerable proportion of patients in this series. This observation likewise warrants cautious interpretation. Adler grading was originally developed as a semiquantitative framework for describing color Doppler vascularity (3) and has not been validated specifically as a diagnostic grading system for cervical G-EAC. Detailed Doppler acquisition parameters were not uniformly retrievable owing to the retrospective design. Adler grade III vascularity in the present series should therefore be regarded as an exploratory sonographic observation rather than a validated diagnostic criterion.

A barrel-shaped cervix has been reported in the imaging literature on gastric-type and minimal deviation cervical adenocarcinoma (15–17). In our cohort, a barrel-shaped cervix was documented in only a few patients; these observations, however, came from pathological or surgical records rather than from a standardized prospective sonographic assessment. This finding should not be interpreted as a sonographic detection rate or as a validated sonographic feature in this study.

### Differential diagnosis and the complementary role of MRI and pathology

Distinguishing G-EAC from benign multicystic cervical lesions is challenging, because both neoplastic and non-neoplastic processes may contain numerous glandular and cystic structures. The relevant differential diagnoses include nabothian cysts, LEGH, minimal deviation adenocarcinoma (MDA), and other cervical adenocarcinomas. MDA is now considered part of the morphological spectrum of gastric-type adenocarcinoma and may show predominantly cystic or multicystic features (6, 15). LEGH, a gastric-type glandular proliferation, may likewise produce multicystic cervical abnormalities that overlap with those seen in gastric-type neoplastic lesions (12–14). Multicystic architecture alone should not be considered diagnostic of malignancy.

MRI can provide complementary information by better characterizing both the distribution of the cystic and solid components and the extent of cervical stromal involvement (12, 14, 16, 17). Earlier MRI studies have examined the characteristic patterns of gastric-type mucin-positive cervical lesions and how these differ from other benign multicystic cervical lesions. The “cosmos pattern,” comprising centrally clustered small cystic and solid components with relatively larger peripheral cysts, has been described in the evaluation of gastric-type mucin-positive cervical lesions (14). This pattern is, however, not specific to invasive G-EAC, because it may also be seen in LEGH, and overlap with other benign cervical cystic lesions has been documented (12–14). MRI findings should therefore be interpreted within the wider clinical and pathological context rather than as a stand-alone diagnostic criterion.

In clinical practice, ultrasound and MRI are best regarded as complementary rather than competing examinations. Ultrasound offers an accessible first assessment of cervical morphology, internal echogenicity, cystic architecture, and vascularity, whereas MRI may further characterize lesion architecture and local extent. Neither ultrasound nor MRI alone, however, can establish a definitive diagnosis of G-EAC, and histopathological examination therefore remains indispensable.

If clinical or imaging findings remain suspicious despite negative cytology or superficial cervical biopsy, adequate tissue should be obtained directly from the endocervical lesion, particularly when superficial specimens are nondiagnostic (8, 9). Because gastric-type lesions may be located deep within the endocervical canal, superficial specimens may not be representative of the underlying lesion.

### Pathological interpretation

Pathological examination remains the cornerstone for the definitive diagnosis of G-EAC. Gastric-type tumors may show heterogeneous morphology and immunophenotypic profiles, so the diagnosis should not rely on a single immunohistochemical marker (6, 9, 18, 19). Immunohistochemical findings should instead be interpreted as supportive evidence alongside the morphology and HPV status. Markers such as MUC6, p16, p53, ER, and PR may aid the pathological assessment, yet their expression can vary among tumors (18). Occasional or focal p16 expression does not in itself establish an HPV-associated origin, and the overall staining pattern should be interpreted together with the tumor morphology and other pathological findings.

Pathological findings in our cohort also revealed substantial local stromal invasion in a considerable proportion of patients with assessable data. Given the small sample and the descriptive design of this study, these findings should not be interpreted as estimates of the population prevalence of deep invasion or as evidence regarding prognosis. They illustrate the extent of disease observed in this particular case series and reinforce the need for adequate pathological assessment once a diagnosis has been established.

Considered together, the clinical, sonographic, MRI, and pathological findings in this series illustrate why G-EAC may be difficult to recognize with any single diagnostic modality. Persistent symptoms, suspicious cervical abnormalities at imaging, and discordance between the clinical findings and negative results on HPV testing, cytology, or superficial biopsy should prompt a careful multidisciplinary evaluation. In this setting, a honeycomb-like sonographic appearance and abundant intralesional vascularity may provide additional imaging clues, yet neither finding should be viewed as specific for G-EAC. The sonographic observations described here are hypothesis-generating, and they require validation in larger prospective controlled cohorts before their diagnostic performance and reproducibility can be firmly established.

### Limitations

Several important limitations of this study should be acknowledged. First, it was a single-center retrospective study with a small sample size (*n* = 13), which may have introduced selection bias and limits the generalizability of the findings. Second, the ultrasound images were reviewed retrospectively by two sonographers who were not blinded to the final histopathological diagnosis, which may have introduced interpretation bias, particularly in the retrospective identification of the honeycomb-like pattern. Third, three different ultrasound systems were used, and detailed Doppler acquisition parameters were not uniformly stored given the retrospective design. In particular, the pulse-repetition frequency, color gain, and wall-filter settings could not be verified retrospectively for every examination. Such technical variation may in turn have affected both the visualization and the grading of intralesional vascularity.

Although Adler grading served as a semiquantitative framework for grading vascularity, it has not been validated specifically for cervical G-EAC. The Doppler findings should therefore be viewed as descriptive and exploratory rather than as validated diagnostic criteria. Preoperative MRI was performed in only 10/13 patients, one of whom also underwent CT, whereas the remaining three patients underwent neither study. Because cross-sectional imaging was not available for all patients, a systematic comparison between ultrasound and MRI or CT was precluded.

No control group of benign multicystic cervical lesions or of other cervical malignancies was included in the present series. The sensitivity, specificity, predictive values, and discriminatory performance of these features therefore could not be assessed. Interobserver agreement was not formally assessed, and the reproducibility of both the honeycomb-like pattern and the vascularity assessment remains uncertain. Future multicenter prospective studies with larger patient cohorts, standardized ultrasound and Doppler protocols, appropriate control groups, blinded image interpretation, and formal interobserver agreement analysis will be required to determine whether these sonographic observations have reproducible diagnostic value.

## Conclusion

Gastric-type endocervical adenocarcinoma is an HPV-independent cervical adenocarcinoma that may be difficult to detect with conventional cervical screening and that may show heterogeneous sonographic appearances. In this series, a honeycomb-like pattern and abundant intralesional vascularity were frequently observed at ultrasound, whereas other lesions appeared as benign cystic lesions or showed no obvious cervical abnormality. These findings may offer useful imaging clues that raise clinical suspicion, yet they should not be regarded as specific or validated diagnostic criteria. Persistent vaginal discharge or abnormal bleeding, as well as suspicious cervical imaging findings, warrant careful clinical evaluation even when HPV testing, cytology, or superficial biopsy are negative. Integrating the clinical findings, ultrasound and complementary MRI, and adequate histopathological assessment may support earlier recognition of G-EAC. Larger prospective controlled studies will be needed to validate both the diagnostic relevance and the reproducibility of these sonographic findings.

## Data Availability

The raw data supporting the conclusions of this article will be made available by the authors, without undue reservation.

## References

[1] Shi H Shao Y Zhang H Ye L Xu E Lu B. Independent validation of distinct clinicopathological features and prognosis among usual-type, mucinous-type and gastric-type endocervical adenocarcinoma categorised by new WHO classification (2020). Pathology. (2022) 54(5):555–62. 10.1016/j.pathol.2021.12.301 35346505

[2] Yasutake N Yokawa Y Tanaka T Mishima R Komamizu M Kuga R. Claudin-18 expression in gastric type adenocarcinoma and HPV-associated adenocarcinoma of the uterine cervix. Histopathology. (2026) 88(5):1003–15. 10.1111/his.70066 41424299 PMC12975664

[3] Adler DD Carson PL Rubin JM Quinn-Reid D. Doppler ultrasound color flow imaging in the study of breast cancer: preliminary findings. Ultrasound Med Biol. (1990) 16(6):553–9. 10.1016/0301-5629(90)90020-D 2238263

[4] Gynecologic Oncology Committee, Chinese Obstetricians and Gynecologists Association, Chinese Medical Doctor Association. Chinese expert consensus on the clinical diagnosis and treatment of gastric-type adenocarcinoma of the uterine cervix (2021 edition). Chin J Pract Gynecol Obstet. (2021) 37(11):1131–6. 10.19538/j.fk2021110112

[5] Kojima A Mikami Y Sudo T Yamaguchi S Kusanagi Y Ito M. Gastric morphology and immunophenotype predict poor outcome in mucinous adenocarcinoma of the uterine cervix. Am J Surg Pathol. (2007) 31(5):664–72. 10.1097/01.pas.0000213434.91868.b0 17460448

[6] Talia KL McCluggage WG. The developing spectrum of gastric-type cervical glandular lesions. Pathology. (2018) 50(2):122–33. 10.1016/j.pathol.2017.09.009 29233547

[7] Cho H Park S Kim HS. Gastric-type endocervical adenocarcinoma: comprehensive cytopathological analysis and comparison with usual-type endocervical adenocarcinoma. In Vivo. (2023) 37(3):1173–81. 10.21873/invivo.13192 37103100 PMC10188018

[8] Chalif J Kistenfeger Q Fulton J Morton M DeVengencie I Weldemichael W. Diagnosis and management of gastric-type endocervical adenocarcinoma: a case report and review of the literature. Gynecol Oncol. (2024) 185:165–72. 10.1016/j.ygyno.2024.02.024 38428332

[9] Turashvili G Morency EG Kracun M DeLair DF Chiang S Soslow RA. Morphologic features of gastric-type cervical adenocarcinoma in small surgical and cytology specimens. Int J Gynecol Pathol. (2019) 38(3):263–75. 10.1097/PGP.0000000000000519 29750702 PMC6230321

[10] Lee JE Chung Y Rhee S Kim TH. Untold story of human cervical cancers: HPV-negative cervical cancer. BMB Rep. (2022) 55(9):429–38. 10.5483/BMBRep.2022.55.9.042 35725012 PMC9537028

[11] Jung H Bae GE Kim HM Kim HS. Clinicopathological and molecular differences between gastric-type mucinous carcinoma and usual-type endocervical adenocarcinoma of the uterine cervix. Cancer Genom Proteom. (2020) 17(5):627–41. 10.21873/cgp.20219 PMC7472441 32859641

[12] Takatsu A Shiozawa T Miyamoto T Kurosawa K Kashima H Yamada T. Preoperative differential diagnosis of minimal deviation adenocarcinoma and lobular endocervical glandular hyperplasia of the uterine cervix: a multicenter study of clinicopathology and magnetic resonance imaging findings. Int J Gynecol Cancer. (2011) 21(7):1287–96. 10.1097/IGC.0b013e31821f746c 21685796

[13] Ando H Miyamoto T Kashima H Takatsu A Ishii K Fujinaga Y. Usefulness of a management protocol for patients with cervical multicystic lesions: a retrospective analysis of 94 cases and the significance of GNAS mutation. J Obstet Gynaecol Res. (2016) 42(11):1588–98. 10.1111/jog.13083 27718288 PMC5108490

[14] Ohya A Kobara H Miyamoto T Komatsu M Shiozawa T Fujinaga Y. Usefulness of the ‘cosmos pattern’ for differentiating between cervical gastric-type mucin-positive lesions and other benign cervical cystic lesions in magnetic resonance images. J Obstet Gynaecol Res. (2021) 47(2):745–56. 10.1111/jog.14602 33331010

[15] Li G Jiang W Gui S Xu C. Minimal deviation adenocarcinoma of the uterine cervix. Int J Gynaecol Obstet. (2010) 110(2):89–92. 10.1016/j.ijgo.2010.03.016 20451906

[16] Kido A Mikami Y Koyama T Kataoka M Shitano F Konishi I. Magnetic resonance appearance of gastric-type adenocarcinoma of the uterine cervix in comparison with that of usual-type endocervical adenocarcinoma: a pitfall of newly described unusual subtype of endocervical adenocarcinoma. Int J Gynecol Cancer. (2014) 24(8):1474–9. 10.1097/IGC.0000000000000229 25188888

[17] Kerwin CM Markese M Moroney MR Smith LP Patel NU. Adenocarcinoma of the uterine cervix, gastric-type (GAS): a review of the literature focused on pathology and multimodality imaging. Abdom Radiol (NY). (2023) 48(2):713–23. 10.1007/s00261-022-03724-w 36334123

[18] Carleton C Hoang L Sah S Kiyokawa T Karamurzin YS Talia KL. A detailed immunohistochemical analysis of a large series of cervical and vaginal gastric-type adenocarcinomas. Am J Surg Pathol. (2016) 40(5):636–44. 10.1097/PAS.0000000000000578 26685087 PMC5564425

[19] Takako K Hoang L Terinte C Pesci A Aviel-Ronen S Alvarado-Cabrero I. Trefoil factor 2 (TFF2) as a surrogate marker for endocervical gastric-type carcinoma. Int J Gynecol Pathol. (2021) 40(1):65–72. 10.1097/PGP.0000000000000680 32897966 PMC7725933

